# Therapeutic Targets for Overcoming BCR::ABL1 Tyrosine Kinase Inhibitor Resistance in Chronic Myeloid Leukemia

**DOI:** 10.32604/or.2025.075217

**Published:** 2026-04-22

**Authors:** Masanobu Tsubaki, Taira Matsuo, Rie Komori

**Affiliations:** Laboratory of Pharmacotherapy, Faculty of Pharmaceutical Sciences at Kagawa Campus, Tokushima Bunri University, 8-53 Hamanocho, Takamatsu, 760-8542, Kagawa, Japan

**Keywords:** Chronic myeloid leukemia, tyrosine kinase inhibitor, resistance, breakpoint cluster region::Abelson murine leukemia 1

## Abstract

Chronic myeloid leukemia (CML) is a hematopoietic malignancy originating from hematopoietic stem cells. It is characterized by the Philadelphia chromosome, which arises from a reciprocal translocation between chromosomes 9 and 22. The breakpoint cluster region::Abelson murine leukemia 1 (BCR::ABL1) fusion protein produced from this chromosome is the main factor responsible for disease onset. Tyrosine kinase inhibitors (TKIs) have led to significant advances in CML treatment and contributed to improved patient survival rates. Nonetheless, a substantial number of patients develop resistance to TKIs, which remains a major challenge in CML therapy. Currently, two mechanisms are considered responsible for TKIs resistance in CML: BCR::ABL1-dependent resistance, involving mutations or overexpression of BCR::ABL1, and BCR::ABL1-independent resistance, which does not depend on BCR::ABL1. This review discusses the recent findings on the resistance mechanisms mediated by BCR::ABL1 mutations. It also focuses on bypass pathways, the B-cell/CLL lymphoma 2 family, tumor suppressor genes, microRNAs, and molecular chaperones as independent resistance mechanisms. Furthermore, the potential for combination therapies targeting these resistance mechanisms is discussed, anticipating further advances in research aimed at overcoming TKI resistance in CML.

## 1 Introduction

Chronic myeloid leukemia (CML) is a hematopoietic malignancy originating from hematopoietic stem cells, with an annual incidence of 1–2 per 100,000 people, accounting for 15% of all leukemia cases [1]. In Western countries, the median age at diagnosis is approximately 65 years, whereas in Asia and Africa, it is less than 50 years [2,3]. CML is characterized by the Philadelphia chromosome, which results from a reciprocal translocation between chromosomes 9 and 22. This chromosome produces the breakpoint cluster region::Abelson murine leukemia 1 (BCR::ABL1) fusion gene. The BCR::ABL1 protein transcribed from this gene possesses constitutive tyrosine kinase activity, which causes CML development. Therefore, BCR::ABL1 is a therapeutic target for CML and serves as a critical factor in its diagnosis and monitoring [4].

Imatinib, the first BCR::ABL1 tyrosine kinase inhibitor (TKI) introduced in the early 2000s, dramatically improved the 5-year and 10-year survival rates compared to conventional interferon or chemotherapy [5–7]. However, despite these therapeutic advances, approximately 20%–30% of patients treated with first-line therapy (first-generation TKI imatinib and second-generation TKIs nilotinib, dasatinib, and bosutinib) develop BCR::ABL1 TKI resistance [3]. BCR::ABL1 TKI resistance mechanisms are broadly categorized into BCR::ABL1-dependent pathways caused by ABL1 gene mutations or overexpression, and BCR::ABL1-independent pathways (activation of bypass pathways, changes in B-cell/CLL lymphoma 2 (Bcl-2) family protein expression, tumor suppressors, microRNAs (miRNAs), and molecular chaperones) [8].

## 2 BCR::ABL1-Dependent Resistance Mechanism

### 2.1 BCR::ABL1 Gene Mutation

**Figure 1:**
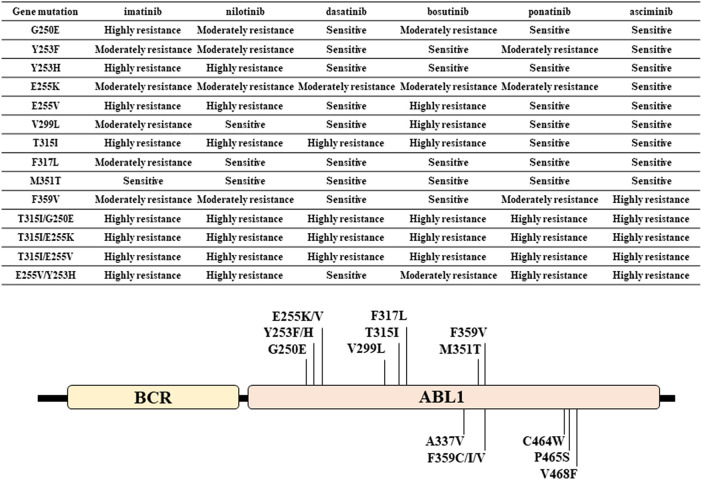
Major ABL1 gene mutations observed in patients with CML and their susceptibility to TKIs. The table in the figure indicates sensitivity, moderate resistance, and high resistance based on the IC50 values for cell proliferation of TKIs in ABL1 mutant cells. The figure shows the location of the ABL1 mutation [10–21]. ABL1: abelson murine leukemia 1; CML: chronic myeloid leukemia; TKIs: tyrosine kinase inhibitors; IC50: 50% inhibition concentration; BCR: breakpoint cluster region

The factors contributing to BCR::ABL1-dependent resistance are mainly attributed to mutations in the BCR::ABL1 gene (Fig. 1). These mutations reduce the efficacy of TKIs by interfering with their binding. Currently, over 100 mutation sites have been identified and susceptibility to TKIs varies depending on the mutation site [9,10]. Representative examples include T315I, a mutation in the binding site of first- and second-generation TKIs; E255K/V and Y253F/H, in the ATP-binding domain; and F359V and M351T, in the catalytic domain [10,11]. Y253F/H, E255K/V, T315I, M351T, and F359V confer resistance to imatinib; whereas Y253H, E255K/V, T315I, and F359V confer resistance to nilotinib; E255K/V and T315I confer resistance to bosutinib; and T315I confers resistance to dasatinib [12–15]. For T315I, ponatinib or asciminib, which bind to the myristoyl pocket of ABL1 and allosterically inhibit BCR::ABL1, were used. The emergence of double mutations, including T315I (T315I/G250E, T315I/E255K, T315I/E255V, and E255V/Y253H) for ponatinib, has been shown to be involved in resistance *in vitro* [16–18]. Furthermore, the dual mutations T315I/E255K has been reported in ponatinib-resistant cases [19,20]. Moreover, V468F, P465S, C464W, F359C/I/V, and A337V are known resistance mutations to asciminib, and V468F, P465S, C464W, and F359I have been identified in clinical cases [21]. As shown in Fig. 1, individual BCR::ABL1 mutations exhibited different susceptibility profiles to various TKIs, necessitating a switch to the TKI corresponding to the mutation. Furthermore, the emergence of genetic mutations other than BCR::ABL1 and chromosomal abnormalities in Ph-positive cells have also been shown to be involved in TKI resistance [22–26]. Therefore, analysis of genetic mutations, not restricted to BCR::ABL1, is crucial for treatment management in CML.

Next-generation sequencing (NGS) has identified ABL1 mutations associated with TKI resistance, as shown in Fig. 1, and genomic abnormalities in genes such as NMT2A, RUNX1, ASXL1, and IKZF1. These genomic abnormalities have been shown to potentially cause drug resistance in CML [27–30]. Furthermore, approximately 15% to 25% of patients exhibit genomic abnormalities other than BCR::ABL1 at CML diagnosis, and these abnormalities have been shown to be associated with TKI failure [28,29]. Moreover, patients with chronic-phase CML who have failed treatment with at least two different TKIs frequently harbor additional oncogenic mutations beyond BCR::ABL1, and that the number of these mutations correlates with an increased risk of TKI treatment failure [31]. Therefore, TKI sensitivity in BCR::ABL1 mutations may be altered by other oncogene mutations. Digital droplet polymerase chain reaction (ddPCR) is a highly sensitive method capable of detecting BCR::ABL1 transcripts, surpassing conventional qualitative PCR. ddPCR exhibits superior specificity and sensitivity compared to NGS in patients with TKI-resistant CML already identified as harboring ABL1 mutations via NGS analysis [32]. Additionally, ddPCR has been shown to detect T315I mutation with high sensitivity in patients with CML [33]. Based on these findings, genomic analysis using NGS and ddPCR may play a crucial role in the management of TKI treatment.

### 2.2 BCR::ABL1 Overexpression

BCR-ABL1 overexpression is a factor in the BCR::ABL1-dependent mechanism of imatinib resistance. However, its frequency is low compared to that of BCR::ABL1 gene mutations, and its significance is low (only two out of 66 imatinib-resistant patients) [34]. Nevertheless, CML cells with high BCR::ABL1 expression exhibited reduced imatinib sensitivity and may promote the emergence of mutant clones compared to low-expression cells. Furthermore, CML cells in the blast crisis phase show higher BCR::ABL1 expression than those in the chronic phase, resulting in lower imatinib sensitivity [34].

## 3 BCR::ABL1-Independent Resistance Mechanisms

The major cause of resistance to BCR::ABL1 TKIs therapy is mutation; however, approximately 40%–50% of cases of resistance to BCR::ABL1 TKIs therapy arise via independent pathways [35,36]. Therefore, elucidating the factors involved in independent pathways may improve the prognosis of patients with drug resistance.

### 3.1 Bypass Pathway

**Figure 2:**
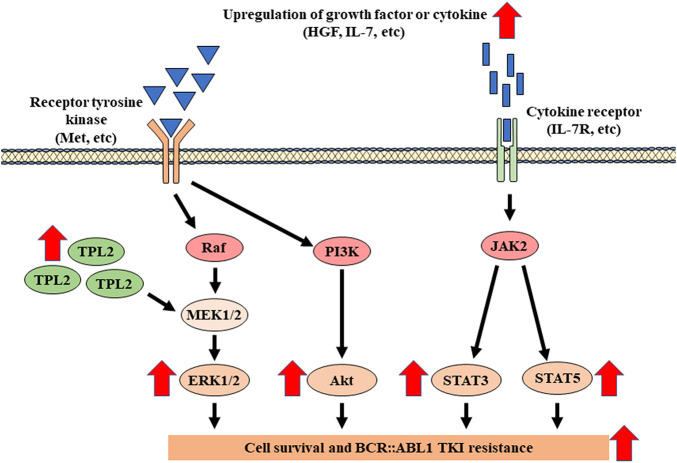
Schematic representation of BCR::ABL1-independent mechanisms via bypass pathways. Activation of downstream signaling pathways (PI3K/AKT, JAK/STAT, and RAS/MAPK) through increased growth factors and cytokines and activation of signaling molecules (receptor tyrosine kinase and TPL2 (MAPKKK)) [41–49]. HGF: hepatocyte growth factor; IL-7: interleukin 7; IL-7R: IL-7 receptor; TPL2: tumor progression locus 2; Raf: rapidly accelerated fibrosarcoma; MEK1/2: mitogen-activated protein kinase kinase 1/2; PI3K: phosphoinositide 3-kinase; JAK2: Janus kinase 2; STAT3: signal transducer and activator of transcription 3; STAT5: signal transducer and activator of transcription 5; BCR: breakpoint cluster region; ABL1: abelson murine leukemia 1; TKI: tyrosine kinase inhibitor

TKI resistance due to the activation of bypass pathways is frequently observed not only in BCR::ABL1 TKIs, but also in other TKI resistance mechanisms [37–40]. BCR::ABL1 TKIs inhibit ABL1 tyrosine kinase activity and induce apoptosis in CML cells by inhibiting the mitogen-activated protein kinase kinase (MEK)/extracellular signal-regulated kinase (ERK), phosphoinositide 3-kinase (PI3K)/Akt, and Janus kinase (JAK)/signal transducer and activator of transcription (STAT) pathways. However, some cells survive by activating these signals through other pathways (Fig. 2). Activation of the MEK/ERK pathway via tumor progression locus 2 (TPL2) overexpression (mitogen-activated protein kinase kinase kinase (MAPKKK)) or the receptor tyrosine kinase MET has been shown to induce imatinib and dasatinib resistance [41–45]. Furthermore, activation of the PI3K/Akt/mammalian target of rapamycin (mTOR) pathway is involved in the acquisition of imatinib resistance *in vitro* and has been observed in patients with imatinib resistance [46]. Moreover, interleukin 7 expressed by bone marrow stromal cells activates JAK/STAT5 in CML cells; STAT3 is activated by the addition of bone marrow stromal cell culture supernatant, and STAT3 activation is observed in patients with imatinib resistance [47–49]. In addition, FLT3, a receptor tyrosine kinase, is activated in approximately half of the acute-phase CML cases and contributes to resistance to BCR::ABL1 TKIs [50]. Activation of the bone morphogenetic pathway receptor (BMPR)/ALK pathway has also been shown to contribute to BCR::ABL1 TKI resistance [51,52]. Thus, various factors contribute to BCR::ABL1 TKI resistance through the activation of bypass pathways, suggesting that treatment targets may be diverse. Consequently, identifying factors that serve as therapeutic targets for each patient using approaches such as NGS-based gene mutation analysis and RNA sequencing is necessary.

### 3.2 Changes in Bcl-2 Family Protein Expression

**Figure 3:**
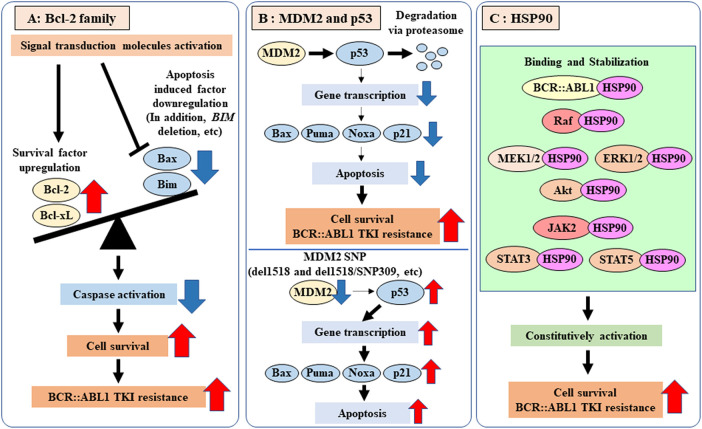
Schematic representation of BCR::ABL1-independent mechanisms mediated by changes in Bcl-2 family, MDM2, p53, and HSP90 expression. (**A**) Activation of signaling molecules increases the expression of Bcl-2 and Bcl-xL, promoting survival and inducing TKI resistance. Additionally, BIM deletion increases the expression of survival factors, such as Bcl-2 [53–56]. (**B**) MDM2 induces TKI resistance by suppressing the expression of Bax and other proteins through the degradation of p53. Furthermore, reduced MDM2 expression due to SNPs increases p53 activity, thereby enhancing TKI sensitivity [58–67]. (**C**) HSP90 induces resistance to TKIs by binding to target proteins (such as signaling molecules), stabilizing them, and inducing their constitutive activation [72–75]. BCR: breakpoint cluster region; ABL1: abelson murine leukemia 1; Bcl-2: B-cell/CLL lymphoma 2; Bcl-xL: B-cell lymphoma extra large; Bax: Bcl-2-associated X protein; Bim: Bcl-2 interacting mediator of cell death; TKI: tyrosine kinase inhibitor; MDM2: mouse double minute protein 2; HSP90: heat shock protein 90; Raf: rapidly accelerated fibrosarcoma; MEK1/2: mitogen-activated protein kinase kinase 1/2; JAK2: Janus kinase 2; STAT3: signal transducer and activator of transcription 3; STAT5: signal transducer and activator of transcription 5

Bcl-2 family proteins are broadly categorized into apoptosis inhibitors (Bcl-2, B-cell lymphoma extra large (Bcl-xL), Myeloid cell leukemia 1 (Mcl-1)) and apoptosis promoters (Bcl-2-associated X protein (Bax), Bcl-2 interacting mediator of cell death (Bim), Noxa, p53 upregulated modulator of apoptosis (Puma), BCL2 associated agonist of cell death (Bad)) (Fig. 3A). Bcl-2 and other proteins regulate Bax function by binding to it [53]. CML patients with *BIM* deletion gene polymorphisms show resistance to BCR::ABL1 TKIs. Furthermore, BH-3 mimetic drugs, such as venetoclax, have been suggested to be potentially effective in these patients [54]. Exosomes secreted by mesenchymal stem cells present in the bone microenvironment increase Bcl-2 expression in CML cells, inducing imatinib resistance [55]. Patients with chronic-phase CML exhibiting imatinib resistance, Bcl-2 overexpression and decreased Bad expression are observed in tumor cells [56]. Furthermore, studies using CRISPR-Cas9 knockout screening to investigate TKI resistance factors have identified the involvement of Bax, Bim, Noxa, and Puma, and have also demonstrated that resistance can be overcome by navitoclax and venetoclax [57]. These findings suggest that changes in Bcl-2 family protein expression regulate intrinsic apoptotic pathways and control TKI-induced cell death. Furthermore, gene deletions or amplifications can be evaluated using NGS.

### 3.3 Altered Expression of Mouse Double Minute Protein 2 (MDM2) and p53

The tumor suppressor gene p53 induced cell death by enhancing the expression of Bax, Puma, Noxa, and others, and suppressed cell proliferation by increasing p21 expression (Fig. 3B). Therefore, disruption of pathways involving this gene promotes cell survival and proliferation [58,59]. Furthermore, p53 loss and factors involved in regulating these tumor suppressor genes are significantly implicated in TKI resistance [60–63].

MDM2 binds to p53, inhibiting its transcriptional activity while also acting as an E3 ubiquitin ligase to promote proteasome-mediated degradation [64]. Furthermore, MDM2 expression increased with CML progression and downregulated p53 expression [65]. BCR::ABL1 increases the expression of p53 and p53 target genes; however, its potent oncogenic effects promote the survival of CML cells [66]. Furthermore, the analysis of BCR::ABL1 TKI efficacy in MDM2 genetic polymorphisms showed that individual MDM2 polymorphisms did not affect TKI sensitivity. However, the *MDM2* single nucleotide polymorphism (SNP) 309 G/G genotype alone was associated with reduced major molecular remission achievement and increased risk of moderate/high Sokal scores, suggesting that it may be a poor prognostic factor. Furthermore, *MDM2* del1518 polymorphism alone, *MDM2* del1518/*MDM2* SNP309, or *MDM2* SNP309/*TP53* SNP215 improved OS in TKI non-responders. Furthermore, TKI non-responders harboring the *MDM2* SNP309/*TP53* SNP215 polymorphism demonstrated a higher molecular response rate, suggesting that this polymorphism may be a low-risk factor in regulating TKI response [67]. These findings suggest that MDM2 polymorphisms may regulate MDM2 expression, which in turn differentially affects p53 activity and potentially contributes to an improved TKI response.

In addition, MDM2 activation is regulated by PAK6, which contributes to TKI resistance by downregulating p53 and p21 expression [68]. MDM2 regulates p21 expression in a p53-independent manner and suppresses cell proliferation even in p53-null CML cells [68]. While p53 deletion has been reported to be involved in imatinib resistance [69], other studies have found no change in p53 gene expression between BCR::ABL1-responders and non-responders [70]. In CML, genomic instability becomes more likely as the disease progresses, and mutations or deletions in p53 are also known to occur [71]. The absence of changes in p53 expression between TKI responders and non-responders may be due to the exclusion of patients with a blast crisis from the study population [70]. However, if MDM2 regulates p21 expression in a p53-independent manner, MDM2 could be a useful therapeutic target for TKI resistance, regardless of the status of the p53 gene in patients with CML.

### 3.4 Heat Shock Protein 90

Heat shock proteins (HSPs) are essential for maintaining the correct folding, stability, and activity of intracellular proteins and play a crucial role in cell survival (Fig. 3C). In tumors, including leukemia, they have been reported to be involved in stabilizing survival-promoting signaling factors and apoptosis-inhibiting factors [72]. HSP90 is involved in the progression and pathogenesis of CML, enhancing the stabilization of BCR::ABL1 by binding to it [73,74]. Furthermore, high expression of HSP90 is involved in imatinib resistance in CML. This factor has also been shown to activate downstream signaling molecules in key signaling pathways downstream of BCR::ABL1, such as the JAK/STAT, PI3K/Akt, and Raf/MEK/ERK pathways, by stabilizing them [72,75].

### 3.5 MicroRNA

MicroRNAs (miRNAs) are non-coding RNA that regulate gene expression by degrading messenger RNA or inhibiting translation. Recently, they have been shown to be involved in the progression of various malignancies, including hematological malignancies. However, miRNAs involved in suppression have also been reported, and oncogenic and tumor-suppressive miRNAs are known [76,77]. In CML, miR-18a-5p high expression is implicated in tumor progression by maintaining JAK2 and phosphorylating STAT3 expression through the downregulation of suppressor of cytokine signaling 5. miR-181a enhances cell proliferation inhibition and imatinib sensitivity by regulating RelA expression, whereas miR-181c expression is lower in resistant patients than in imatinib-sensitive patients. miR-30a and miR-30e expression is downregulated in patients with CML, and their upregulation increases imatinib sensitivity. miR-21 and miR-155 are upregulated in patients with CML and are involved in disease progression and imatinib resistance [78]. While miR-29 has been reported to act as an oncogene in acute myeloid leukemia and contribute to imatinib resistance by regulating ten-eleven translocation (TET) and neurofibromatosis type 1 (NF1) expression, it has also been shown to suppress CML cell proliferation by targeting ABL1 [79–82]. Furthermore, while some reports have indicated that miR-10 expression is elevated in imatinib-resistant cells, others have shown that it is decreased [83–85]. Thus, miRNAs are potential therapeutic targets for CML because of their involvement in tumor progression and suppression. However, a detailed investigation is required to elucidate the roles of certain miRNAs, including the causes of resistance and their impact on leukemia stem cells.

## 4 Therapeutic Approaches to BCR::ABL1 TKIs Resistance

For treating resistance to BCR::ABL1 TKIs, therapeutic approaches tailored to each cause are crucial. The confirmation of TKI resistance or recurrence is also an important factor in treatment decisions. Essential aspects of CML treatment management include confirming ABL1 mutations via NGS, detecting other cancer gene mutations, and verifying ABL1 gene mutations and BCR::ABL1 transcript levels using ddPCR [27,32,33,86].

### 4.1 Therapeutic Approaches to Resistance Due to Genetic Mutations

Currently, imatinib, nilotinib, dasatinib, and bosutinib are first-line treatment options in many countries. When resistance mutations arise, ponatinib and asciminib become appropriate options. Asciminib resistance arises from mutations, such as V468F, P465S, C464W, F359C/I/V, and A337V, in the myristoyl pocket of its binding site. *In vitro* and *in vivo* analyses suggested that ponatinib monotherapy and the combination of ponatinib and venetoclax might be effective against resistant clones harboring these mutations [87]. Furthermore, based on a case report, dasatinib has been shown to be effective against A337V mutations [88]. Although dual mutations cause ponatinib resistance, the combination of asciminib and ponatinib induces cell death in resistant cells derived from patients with CML and suppress tumor growth *in vivo* [18]. Furthermore, the combination of ponatinib and asciminib has been reported to be potentially effective against the T315I/E335G double mutation [89]. Furthermore, in clinical trials, the third-generation BCR::ABL1 TKI olverembatinib achieved a complete cytogenetic response in 15 of 26 patients who had received prior ponatinib treatment, and a major molecular response in 11 of 30 patients. Among the patients who received asciminib treatment, four of eight achieved a complete cytogenetic response and four of twelve achieved a major molecular response [90]. Furthermore, clinical trials combining asciminib with imatinib, nilotinib, or dasatinib have attempted to prevent dual mutations and resistance, and favorable results have been reported [91].

Allogeneic hematopoietic stem cell transplantation (HSCT) has become a treatment option for BCR::ABL1 TKIs (particularly in patients who have failed three or more TKIs and/or ponatinib/asciminib) and persistent CML cells with disease progression [92]. The 2020 European Leukemia Network and National Comprehensive Cancer Network guidelines also present HSCT as an effective treatment option for patients with acquired resistance to TKIs (both BCR::ABL1-dependent and independent mechanisms) [3,93]. Seventy patients who underwent HSCT after BCR::ABL1 TKI failure for 20 years had a 5-year overall survival rate of 57.7% (45.1%–68.5%). It also indicated that patients who achieved cytogenetic and molecular remission prior to transplantation had a better prognosis than those who achieved only hematological remission [94]. Furthermore, HSCT performed more than one year after diagnosis increases the risk of non-relapse mortality (NRM) by approximately 52% compared with earlier timing. Moreover, receiving treatment after initiating TKI therapy significantly reduces the risk of NRM by up to 40% compared with treatment before TKI initiation [95]. However, if recurrence occurs after HSCT, treatment primarily involves interferons and chemotherapy, and the prognosis is extremely poor. Therefore, regular long-term monitoring of BCR::ABL1 transcripts is necessary after HSCT [96].

### 4.2 Therapeutic Approaches to Resistance via Bypass Pathway Activation

Resistance due to bypass pathway activation is difficult to interpret because its causes are diverse; however, it is a major factor in BCR::ABL1-independent resistance. Some clinical trials have been conducted based on the basic research findings. JAK2-mediated STAT3 and STAT5 activation has been shown to confers TKI resistance to CD34-positive CML cells [97]. Therefore, several clinical trials combining JAK inhibitors with TKIs have been conducted. Combination therapy with ruxolitinib and nilotinib demonstrated higher efficacy than nilotinib alone, with no issues regarding tolerability [98]. Furthermore, a Phase 2 trial reported that ruxolitinib plus TKI combination therapy demonstrated high efficacy with no tolerability issues compared with TKI monotherapy [99]. Additional clinical trials (NCT03654768 and NCT03610971) are currently ongoing. Additionally, peroxisome proliferator-activated receptor γ (PPARγ) is known to negatively regulate STAT5 expression, and pioglitazone, a PPARγ agonist, enhances the effect of imatinib by decreasing STAT5 expression [100]. In a clinical trial involving patients who had not achieved molecular response 4.5 (BCR-ABL1/ABL1^IS^ RNA levels ≤ 0.0032%), combination therapy with imatinib and pioglitazone demonstrated a favorable cumulative achievement rate of 56% for molecular response 4.5 over 12 months, compared to 23% with imatinib alone, with no issues regarding tolerability [101].

Basic research has also demonstrated that the mTOR inhibitors rapamycin and everolimus enhance the effects of imatinib, inducing cell death in imatinib-resistant cells [102–104]. Clinical trials (NCT00093639 and NCT00101088) have been conducted in patients with CML; however, the detailed results remain unclear. Targeting mTOR may be effective; however, more effective agents are required. Additionally, basic research has revealed that the activation of the MEK/ERK pathway is also important in BCR::ABL1-independent resistance; however, no clinical trials have been conducted. Moreover, in basic research, the dual inhibitor KF1601, which targets both BCR::ABL1 and FLT3, induces cell death in acute-phase CML cells harboring the BCR::ABL1 T315I mutation and FLT3 activation [105]; combined therapy with ALK TKIs suppressing BMPR/ALK pathway activation and BCR::ABL1 TKIs may also be effective [106]. In the future, agents that inhibit this pathway are expected to serve as therapeutic tools for overcoming resistance.

### 4.3 Therapeutic Approach Targeting Resistance via Changes in Bcl-2 Family Protein Expression

Bcl-2 has been shown to be highly expressed in cells during the acute phase of CML, and it may contribute to TKI resistance [107]. Combination therapy with nilotinib and venetoclax in CD34-positive cells isolated from patients with CML has been shown to enhance apoptosis induction compared to monotherapy with either agent [108]. Furthermore, the combination of nilotinib and venetoclax induces significantly more apoptosis in CML cells collected from patients with acute-phase CML than in bone marrow cells collected from healthy donors [109]. Moreover, the combination of ponatinib and venetoclax has been shown to suppress tumor growth and prolong survival *in vivo* in the asciminib-resistant CML cell line KCL-22 [87]. The efficacy of BCR::ABL1 TKIs + venetoclax combination therapy has been evaluated in patients with acute-phase CML, with a reported response rate of 75% [110]. Furthermore, clinical trials (NCT02689440 and NCT04188405) are currently underway to evaluate combination therapy with dasatinib and venetoclax in patients with early chronic-phase CML and combination therapy with decitabine, venetoclax, and ponatinib in patients with acute- or accelerated-phase CML, with anticipated promising results.

### 4.4 Therapeutic Approaches Targeting Resistance via MDM2 and p53 Expression Changes

Stabilization of p53 by MDM2 inhibitors, such as Nutlin-3 and MI-219, enhances imatinib sensitivity in CML cells with or without BCR::ABL1 mutations by increasing the expression of p53 target genes, thereby inducing apoptosis [111,112]. Furthermore, p53 activation via MDM2 inhibition eliminates the pluripotency of CML stem cells and potentially overcomes BCR::ABL1 TKI resistance [113]. MDM2 inhibitors are ineffective in treating TP53-mutated malignancies. Clinical trials have evaluated the efficacy of KRT-232 in combination with either nilotinib or dasatinib in patients with p53 wild-type chronic-phase or accelerated-phase CML who are resistant or refractory to at least one BCR::ABL1 TKI (NCT04835584).

### 4.5 Therapeutic Approach Targeting Resistance via HSP90

Inhibition of HSP90 induces degradation through destabilization of BCR::ABL1 and downstream signaling factors, such as Raf-1 and Akt, making it a promising therapeutic approach for both BCR::ABL1-dependent and independent resistance. *In vivo* studies have demonstrated that HSP90 inhibition suppresses tumor growth and prolongs survival in T315I-positive CML cells, while also reducing the number of CML stem cells [114]. Furthermore, the HSP90 inhibitor NVP-AUY922 induces cell death in cells harboring various ABL1 mutations responsible for BCR::ABL1 inhibitor resistance when combined with imatinib or nilotinib [115]. Moreover, HSP90 inhibitors have been reported to be effective against imatinib resistance caused by the activation of bypass pathways such as Met receptor tyrosine kinase activation [74]. Currently, the only approved HSP90 inhibitor is pimitespib; however, this drug is indicated for gastrointestinal stromal tumors and cannot be used for CML treatment. Gamitrinib has been studied for advanced cancer (NCT04827810), ganetespib for hormone receptor-positive breast cancer (NCT01560416), and XL888 in combination with vemurafenib and cobimetinib for BRAF-mutated melanoma (NCT02721459). If these clinical trials yield useful results, they could expand the treatment options and potentially be applied to CML therapy.

### 4.6 Therapeutic Approach to Resistance via miRNA

CML stem cells from patients with chronic-phase CML show universally reduced miRNA levels compared to hematopoietic stem cells from healthy donors, suggesting their involvement in CML progression, treatment responsiveness, and TKI resistance [116,117]. Furthermore, miRNAs such as miR-30 and miR-185 are involved in the degradation of BCR::ABL1 mRNA in TKI-resistant cells, suggesting their potential as therapeutic targets for overcoming drug resistance [117]. However, while miR-29 suppresses CML cell proliferation by targeting ABL1, acting as a therapeutic and favorable prognostic factor, it also acts as an oncogene in acute myeloid leukemia by regulating TET expression, contributing to proliferation and imatinib resistance by regulating NF1 expression [79–82]. Furthermore, while some reports indicate that miR-10 is downregulated in imatinib resistance and is involved in proliferation suppression, others suggest that its upregulation contributes to imatinib insensitivity [83–85]. Although miRNAs have great potential for elucidating disease mechanisms and as therapeutic tools, further research is essential to translate these findings into effective treatment strategies.

## 5 Conclusion

Recent studies have elucidated key cellular and molecular pathways in CML and have presented novel therapeutic strategies for targeting these pathways. Although TKIs have significantly advanced CML treatment, resistance and insensitivity remain as challenges, making the exploration of approaches to overcome these critical issues. Novel approaches, including those involving miRNAs, have the potential to improve TKI sensitivity, overcome resistance, and preventing resistance development. A comprehensive multitarget strategy is essential to address the complexity of CML progression and resistance. The evolving landscape of CML treatment relies on research by many scientists, driving the discovery of innovative strategies, including mechanisms to circumvent BCR::ABL-independent resistance. Although many of these approaches remain experimental, further research and clinical validation are crucial for translating these findings into effective treatment options.

## Data Availability

Not applicable.

## Abbreviations

Bad: Bcl2 associated agonist of cell death
Bax: Bcl-2-associated X protein
Bcl-2: B-cell/CLL lymphoma 2
Bcl-xL: B-cell lymphoma extra large
BCR::ABL1: Breakpoint cluster region::Abelson murine leukemia 1
Bim: Bcl-2 interacting mediator of cell death
BMPR: Bone morphogenetic pathway receptor
CML: Chronic myeloid leukemia
ddPCR: Digital droplet polymerase chain reaction
ERK: Extracellular signal-regulated kinase
HSCT: Hematopoietic stem cell transplantation
HSP: Heat shock protein
JAK: Janus kinase
MAPKKK: Mitogen-activated protein kinase kinase kinase
Mcl-1: Myeloid cell leukemia 1
MDM2: Mouse double minute protein 2
MEK: Mitogen-activated protein kinase kinase
miRNA: MicroRNA
mTOR: Mammalian target of rapamycin
NGS: Next-generation sequencing
NRM: Non-relapse mortality
PI3K: Phosphoinositide 3-kinase
PPARγ: Peroxisome proliferator-activated receptor γ
Puma: p53 upregulated modulator of apoptosis
SNP: Single nucleotide polymorphism
STAT: Signal transducer and activator of transcription
TKI: Tyrosine kinase inhibitor
TPL2: Tumor progression locus 2
